# Prenatal Hypoxia Induces Cl^–^ Cotransporters KCC2 and NKCC1 Developmental Abnormality and Disturbs the Influence of GABA_A_ and Glycine Receptors on Fictive Breathing in a Newborn Rat

**DOI:** 10.3389/fphys.2022.786714

**Published:** 2022-02-16

**Authors:** Céline Caravagna, Alexis Casciato, Jacques-Olivier Coq, Sylvie Liabeuf, Cécile Brocard, Julie Peyronnet, Laurence Bodineau, Florence Cayetanot

**Affiliations:** ^1^Department of Neurology, F.M. Kirby Neurobiology Center, Boston Children’s Hospital, and Harvard Medical School, Boston, MA, United States; ^2^Sorbonne Université, Inserm UMR_S1158, Neurophysiologie Respiratoire Expérimentale et Clinique, Faculté de Médecine Site Pitié-Salpétrière, Paris, France; ^3^Institut de Neurosciences de la Timone, UMR 7289, CNRS, Aix-Marseille Université, Marseille, France

**Keywords:** prenatal hypoxia, newborn, breathing, NKCC1, KCC2

## Abstract

Prenatal hypoxia is a recognised risk factor for neurodevelopmental disorders associated with both membrane proteins involved in neuron homeostasis, e.g., chloride (Cl^–^) cotransporters, and alterations in brain neurotransmitter systems, e.g., catecholamines, dopamine, and GABA. Our study aimed to determine whether prenatal hypoxia alters central respiratory drive by disrupting the development of Cl^–^ cotransporters KCC2 and NKCC1. Cl^–^ homeostasis seems critical for the strength and efficiency of inhibition mediated by GABA_A_ and glycine receptors within the respiratory network, and we searched for alterations of GABAergic and glycinergic respiratory influences after prenatal hypoxia. We measured fictive breathing from brainstem in *ex vivo* preparations during pharmacological blockade of KCC2 and NKCC1 Cl^–^ cotransporters, GABA_A_, and glycine receptors. We also evaluated the membrane expression of Cl^–^ cotransporters in the brainstem by Western blot and the expression of Cl^–^ cotransporter regulators brain-derived neurotrophic factor (BDNF) and calpain. First, pharmacological experiments showed that prenatal hypoxia altered the regulation of fictive breathing by NKCC1 and KCC2 Cl^–^ cotransporters, GABA/GABA_A_, and glycin. NKCC1 inhibition decreased fictive breathing at birth in control mice while it decreased at 4 days after birth in pups exposed to prenatal hypoxia. On the other hand, inhibition of KCC2 decreased fictive breathing 4 days after birth in control mice without any change in prenatal hypoxia pups. The GABAergic system appeared to be more effective in prenatal hypoxic pups whereas the glycinergic system increased its effectiveness later. Second, we observed a decrease in the expression of the Cl^–^ cotransporter KCC2, and a decrease with age in NKCC1, as well as an increase in the expression of BDNF and calpain after prenatal hypoxia exposure. Altogether, our data support the idea that prenatal hypoxia alters the functioning of GABA_A_ and glycinergic systems in the respiratory network by disrupting maturation of Cl^–^ homeostasis, thereby contributing to long-term effects by disrupting ventilation.

## Introduction

Development of a mammal neural system is influenced by early life experiences. According to the hypothesis of “developmental programming of health and disease” or “fetal origins of disorder later in life,” maternal environment has an impact on foetal development, and even on adult health (Barker et al., 1993; de Boo and Harding, 2006; Li et al., 2012). Foetal growth and development are constrained by oxygen limitations due to maternal environment including high altitude and pathological situations, such as maternal severe pulmonary and cardiac diseases, chronic anaemia, and intrauterine perfusion (Brodsky and Christou, 2004). Prenatal hypoxia has indeed been characterised as a major cause of neurodevelopmental disorders that can lead to chronic neurological disabilities in children (Baud et al., 2004; Golan and Huleihel, 2006). Related to one of the earliest motor behaviours to develop in foetal state, breathing is sensitive to early prenatal hypoxic stress that induces short- and long-term effects in breathing (Kobayashi et al., 1985; Peyronnet et al., 2000; Powell, 2007; Tree et al., 2016).

Breathing depends on a central respiratory drive (CRD) from the brainstem neuronal network in which neural groups interact with several neurotransmitters such as GABA and glycine (Onimaru et al., 1990; Wittmeier et al., 2008; Koizumi et al., 2013; Richter and Smith, 2014; Anderson et al., 2016). GABA and glycine modulate the central respiratory drive by acting on Cl^–^ channel-receptors, i.e., GABA_A_ and glycine receptors. In immature neurons, limited expression of the K^+^/Cl^–^ cotransporter (KCC2) that extrudes Cl^–^ from cells and greater expression of the Na^+^/K^+^/Cl^–^ cotransporter (NKCC1), which intrudes Cl^–^ into cells lead to high intracellular Cl^–^ concentrations (Blaesse et al., 2009). In such conditions, the activation of GABA_A_ and glycine receptors induces an increase in Cl^–^ outward conductance that depolarises neurons and therefore promotes excitation. In mature neurons, the membrane expression of KCC2 increases whereas the NKCC1 expression decreases, thus leading to a weak intracellular Cl^–^ concentration (Liu and Wong-Riley, 2012). In this condition, the activation of GABA_A_ and/or glycine receptors induces an increase in Cl^–^ inward conductance that hyperpolarises neurons that promote inhibition (Rivera et al., 2004; Vinay and Jean-Xavier, 2008; Stil et al., 2009). The expression of KCC2 and NKCC1 has several regulators including brain-derived neurotrophic factor (BDNF), calpain, insulin growth factor, and prenatal hypoxia may up or downregulate the expression or activation of these regulators (Kelsch et al., 2001; Watanabe and Fukuda, 2015). As reported in perinatal periods, CRD appears strongly influenced by the functional status of Cl^–^ cotransporters. For instance, KCC2-deficent mice die quickly after birth due to a respiratory drive failure, and KCC2 regulates rhythmic respiratory-related activity of hypoglossal nuclei (Hubner and Stein, 2001; Okabe et al., 2015). As Cl^–^ homeostasis depends on environmental conditions including prenatal stress, we hypothesised that a defect in Cl^–^ cotransporters disrupts GABA modulation of breathing, as observed after a gestational stress (Delhaes et al., 2014; Pozzi et al., 2020).

In the present study, we examined how changes in the expression of KCC2, NKCC1, GABA/GABA_*A*,_ and glycine influence CRD at birth by using a model of prenatal hypoxia that also reproduces intrauterine growth restriction (Baud et al., 2004; Pham et al., 2015; Tree et al., 2016). We hypothesise that prenatal hypoxia causes reduced expression of Cl^–^ cotransporters KCC2 and NKCC1, thus leading to disturbances in respiratory frequency, through abnormal shifts toward depolarisation or hyperpolarisation after activation of GABA_A_ and glycine receptors. To determine the impact of chronic prenatal hypoxia, pharmacological studies were combined to electrophysiological recordings in *en bloc* preparations from newborn rats. We also quantified the membrane expression of the two Cl^–^ cotransporters in the brainstem using Western Blot and underlying contributors in their regulation, BDNF by ELISA, and calpain by immunohistochemistry.

## Materials and Methods

All experimental procedures were approved by the “INT Neurosciences Ethic Committee No. 71 for Animal Research” in Marseille (national number of ethical agreement is C1305518) and were performed in accordance with Directive 2010/63/EU of the European Parliament and the Council of September 22, 2010 and French law (2013/118). All efforts were made to minimise the number of animals used and their suffering.

### Animals and Chronic Gestational Hypoxia Model

Sprague–Dawley pregnant rats (Charles River, France) were housed with food and water *ad libitum* in a 12-h light/dark cycle. Pregnant rats were exposed to hypoxia (10% O_2_/90% N_2_) from day 5 to day 20 of gestation, as previously described (Peyronnet et al., 2000). On day 20, they were housed individually and birth occurred in normoxia. Newborn rats grew up in normoxia and constituted the prenatal hypoxia group [prenatal hypoxia (PH); *n* = 151]. A control group of newborn rats was composed of pups from pregnant rats whose gestation occurred under normoxic conditions [21% O_2_; normoxic prenatal group, normoxic prenatal (CONT); *n* = 162]. This high number of animals is because we have 4 drugs applied on 8 distinct groups: PH and CONT, two ages, medullary-spinal cord (MS)/ponto-medullary-spinal cord (PMS) preparations for electrophysiological experiments (*n* = 7–12 per test), Western blot (*n* = 6–8 per group), and Elisa test quantification (*n* = 5 per group).

### Electrophysiological Recordings in *ex vivo* Central Nervous System Preparations

Experiments were conducted on the medullary–spinal cord (MS) and the ponto–medullary–spinal cord (PMS) preparations isolated under deep cold anesthesia by immersion in ice water from newborn rats from the day of birth (counted as P0) to fourth postnatal day (P4) (Okada et al., 1998; Rousseau and Caravagna, 2015; Tree et al., 2016). Two groups of age were distinguished to assess central breathing network maturation: P0-1 and P3-4. MS and PMS preparations differed only in the rostral cut: at the level of anterior inferior cerebellar arteries, caudal to the VIII cranial nerve exit points for MS preparation, rostral to the fifth cranial nerves at the level of superior cerebellar arteries, and caudal edge of inferior colliculi for PMS preparations. A caudal section was done between the seventh and eighth cervical spinal roots. *Ex vivo* preparations were rapidly dissected out and placed in a recording chamber (5 mL), ventral surface upward. This chamber was continuously superfused (5 mL per min) with artificial cerebro-spinal fluid (a-CSF) maintained at 27°C ± 1°C (pH 7.4) and bubbled with carbogen (95%O_2_/5%CO_2_). The a-CSF composition was (in mM): 129.0 NaCl, 3.35 KCl, 21.0 NaHCO_3_, 1.26 CaCl_2_, 1.15 MgCl_2_, 0.58 NaH_2_PO_4,_ and 30.0 D-glucose. The fourth cervical ventral root (C4) was sucked into a glass micropipette and its electrical activity was filtered (10–3,000 Hz), amplified (5,000×), integrated (time constant 100 ms), and digitised (sampling frequency, 5,000 Hz) by using Spike 2 data analysis system (Cambridge Electronik design, United Kingdom). The frequency of C4 burst discharges was considered as the fictive respiratory frequency (fR, expressed in cycles per minute, c.min^–1^). fR was averaged over 5-min periods regardless of the experimental condition. After the surgical procedure, preparations were superfused for 20 min with a-CSF until stable rhythmic C4 discharges were recorded. During such stable conditions, the collected fR is considered as “basal condition” or predrug values.

### Pharmacological Experiments

After determination of basal fR, the control a-CSF (i.e., a-CSF without drugs) was then replaced by a-CSF added with one of the following drugs: bumetanide (10 μM; inhibitor of NKCC1); VU0240551 (10 μM, selective KCC2 inhibitor); picrotoxin (20 μM; GABA_A_ antagonist); strychnin (1 μM; glycine receptor antagonist) for a test period of 20 min. These applications were followed by a recovery period (20 min) under control a-CSF. Drug concentrations have been established according to the literature (Iizuka, 2003; Ren and Greer, 2006), except for VU0240551, which was tested at 1, 5, 10, 25, and 50 μM. Thus, we used 10 μM of VU0240551 to determine the lowest concentration necessary to change fictive breathing in CONT pups. All drugs purchased at Sigma (Sigma-Aldrich, Saint-Quentin-Fallavier, France) were directly dissolved in a-CSF, except for VU0240551, which was previously dissolved in a-CSF that included 0.0025% DMSO.

### Western Blot

P0-1 and P3-4 pups were killed by decapitation after deep-cold anesthesia and quickly dissected to search for quantitative expression of NKCC1 and KCC2 at the membrane (Danneman and Mandrell, 1997). The pons was quickly frozen in liquid nitrogen and stored at −80°C. Supernatant protein concentrations were determined with a detergent-compatible (DC) protein assay (Bio-Rad). Briefly, samples were homogenised in cold lysis buffer A and centrifuged at 18,000 *g* for 30 min at 4°C. Samples were then homogenised in cold lysis buffer B without detergent and centrifuged. Pellets were collected in lysis buffer A without DTT. KCC2 or NKCC1 were then immunoprecipitated, separated in 7% SDS-PAGE, and transferred to a polyvinylidene fluoride membrane. After blockade in Tris-buffered saline plus 5% non-fat dry milk, membranes were exposed overnight at 4°C to a polyclonal rabbit KCC2-specific antibody (07-432; MERCK Millipore) diluted 1:500 or a monoclonal NKCC1-specific antibody (clone T4, Developmental Studies Hybridoma bank) diluted 1/500 in the blocking solution. ImmunoPure goat-horseradish-peroxidase–conjugated rabbit or mouse-specific antibody (1:500 in blocking solution, 1 h at 22°C) was used for chemiluminescent detection (Pierce Biotech). Signal intensities were measured with the image analysis software Quantity-One (Bio-Rad).

### ELISA Test

P3-4 aged pups were killed by decapitation after freezing with anaesthesia and quickly dissected out. Pons was isolated, quickly frozen in liquid nitrogen, and then conserved at −80°C. Pons were homogenised in cold RIPA lysis buffer (25 mM Tris–HCl pH7.6, 150 mM NaCl, 1% NP-40, 1% sodium deoxycholate, 0.1%SDS; Pierce Biotechnology, Rockford, IL, United States) supplemented with protease and phosphatase inhibitors cocktail (Sigma-Aldrich, France). Samples were then centrifuged at 15,000 *g* for 30 min at 4°C. Total protein concentration of each sample was determined by BCA protein assay kit (Pierce Biotechnology, Rockford, IL, United States) and adjusted to 80 μg/mL. BDNF Emax immunoassay system (Promega, France) was performed according to the manufacturer’s instructions. BDNF concentrations were expressed as picograms per mL.

### Immunohistochemistry

To identify prenatal hypoxia-induced changes in the expression of calpain 1, immunohistochemical detection was performed on PH (*n* = 5) and CONT (*n* = 5) newborn rats at P3-P4. Newborn rats were anaesthetised with deep cold anesthesia by immersion in ice water (Danneman and Mandrell, 1997). A block containing pons and medulla oblongata was dissected out from the brain and then fixed by immersion in a fixative solution containing 4% paraformaldehyde in phosphate-buffered saline (PBS; 0.1 m, pH 7.4) for 48 h and then stored for 24 h in a cryoprotectant solution containing 30% sucrose in phosphate buffer at pH 7.4. Serial coronal sections through the pons and medulla oblongata of all pups were cut on a freezing microtome (Leica CM 1510S) at a thickness of 30 μm. Immunohistochemical detection of calpain 1 was processed on free-floating coronal sections. Sections were first incubated with a mouse monoclonal antibody against calpain 1 (sc-271313; Santa Cruz Biotechnology Inc., CA, United States, 1:2,000 in 1% bovine serum albumin, BSA; 48 h, 4°C) and then with a biotinylated horse anti-mouse antibody (BA-2000, Vector Laboratories, Burlington, ON, Canada; 1/500; 2 h). Calpain 1 was finally identified by using a NovaRed Kit (Vector NovaRED Substrate Kit, Vector Laboratories, Burlington, ON, Canada), which resulted in a red/brown coloration. Antibodies specificity was verified with the omission of primary or secondary antibodies in some sections. No labelling was observed in these sections. All the sections were mounted in sequential caudo-rostral order on silanised slides, air-dried, and coverslipped with EUKITT (Bio Optica, Milan, Italy). Using a Leica microscope (Leica DM 2000; Leica Microsystems, Heidelberg, Germany) with bright-field illumination, representative images were photographed at 10× or 20× magnification with a digital camera (Leica DFC450C, Leica Microsystems, Heidelberg, Germany). Calpain-positive cells were visually counted in each section collected through the respiratory related area of the pons, and therefore throughout the rostrocaudal extent of each nucleus, using a Leica microscope (Leica DM2000; Leica Microsystems, Heidelberg, Germany). We analysed the number of calpain positive cells in locus coeruleus, sub-coeruleus nucleus, lateral and medial parabrachial nuclei, and Kolliker füse nucleus. The mean number of calpain-positive cells per section was calculated for each nucleus in each animal.

### Statistics

Data were analysed with GraphPad (GraphPad Prism8, San Diego, CA, United States). Normality of data distribution was assessed using d’Agostino and Pearson normality test. Results were given as mean ± SEM when data complied to normality or as median and interquartile range [Q1; Q3] when normality was not detected. Statistical analyses of electrophysiological data were performed on data averaged over a 5-min time-period. The value of fR was expressed in absolute values (c.min^–1^) in the text. Statistical comparisons of body weight between CONT and PH pups were assessed using a Mann and Whitney test. Statistical comparisons of fR between basal conditions and drug challenges for all groups (MS or PMS, P0-1 or P3-4, CONT or PH) were assessed with one-way repeated measures analysis of variance (ANOVA) followed by the *post hoc* Holm-Sidak’s multiple comparison test or with Friedman test followed by the *post hoc* Dunn’s multiple comparison. The influence of age and/or prenatal hypoxia on fR was assessed using a two-way analysis of variance (ANOVA) test followed by the *post hoc* two-stage linear step-up procedure of Benjamini, Krieger, and Yekutieli. To avoid differences related to basal changes in fR caused by the presence or absence of the pons or by differences in age, the comparison of fR between CONT and PH pups during drug challenges was done on normalised fR (expressed in percentage of pre-drug values of fR). Comparisons of the effect on fR between PH and CONT at two different age groups within the last 5 min of each pharmacological test were done using two-way analysis of variance (ANOVA) test followed by the *post hoc* two-stage linear step-up procedure of Benjamini, Krieger, and Yekutieli to decipher an age and/or prenatal hypoxia effect on fR. For Western blot analysis, comparisons of KCC2 and NKCC1 expression and the ratio NKCC1/KCC2 between PH and CONT and P0 and P4, respectively, were done using a Kruskal–Wallis test followed by the *post hoc* two-stage linear step-up procedure of Benjamini, Krieger, and Yekutieli. For ELISA test and immunohistochemistry, CONT group was compared to PH group for each age set using Mann and Whitney test. Level of statistical significance was set at *p* < 0.05.

## Results

Prenatal hypoxia leads to a decrease in body weight. At P0-1, we noted a lower weight (*p* = 0.006) in the PH group (6.18 ± 0.09 g; *n* = 75) in comparison with CONT group (6.56 ± 0.07 g; *n* = 85). At P3-4, this lower body weight persisted (*p* = 0.027) with mean weights of 8.96 ± 0.17 and 9.56 ± 0.17 in PH (*n* = 76) and CONT (*n* = 77) groups, respectively. Age influenced fR in *ex vivo* preparations. Indeed, in MS preparations, fR increasesd with age in CONT pups (*p* = 0.0002) without there being any change in PH pups, whereas in PMS preparations, fR increased in PH pups (*p* = 0.0002) without there being any change in CONT groups. Prenatal hypoxia influenced fR in *ex vivo* preparations. Indeed, at P0-1, fR was higher in PH pups than in CONT pups in MS preparations (*p* = 0.036), whereas in presence of the pons, fR was lower in PH pups than in the CONT group (*p* = 0.025).

### Abnormalities in Fictive Breathing Modulation by NKCC1 Induced by Prenatal Hypoxia

#### P0-1

**FIGURE 1:**
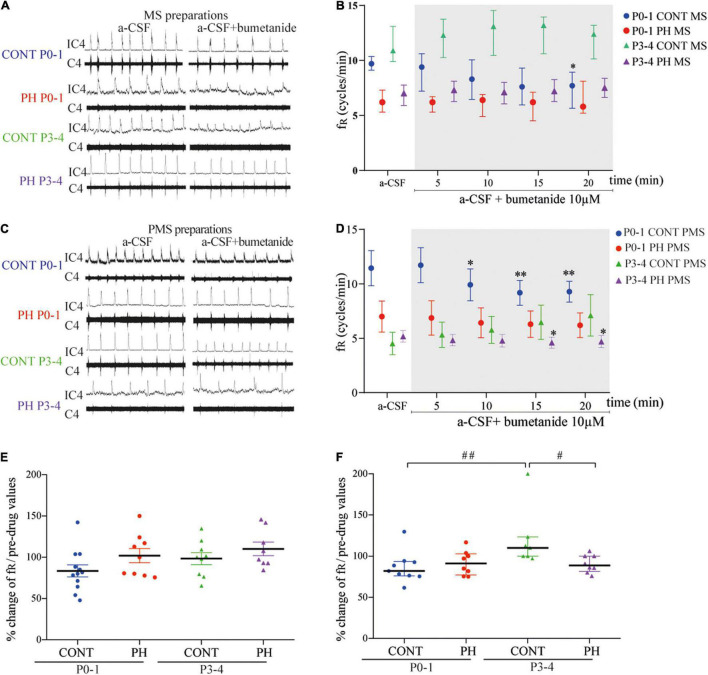
NKCC1 inhibition in the pons altered fictive breathing. **(A,C)** Representative recordings in the medulla **(A)** and ponto-medullary **(C)** preparations before (a-CSF) and during (a-CSF + bumetanide) bumetanide application, in controls (CONT) and prenatal hypoxic (PH) pups, at P0-1 and P3-4. **(B,D)** Frequency of fictive breathing before (a-CSF) and every 5 min during (a-CSF + bumetanide 10 μM) bumetanide application, in CONT and PH pups, at P0-1 and P3-4. The fR is expressed as median [Q1; Q3] in medulla (MS) and as mean ± SEM in ponto-medullary (PMS) preparations. **(E,F)** Scatter plots with surimposed mean ± SEM in medulla **(E)** and median [Q1; Q3] in ponto-medullary **(F)** preparations, illustrating changes in fR during the last 5 min of bumetanide exposure, expressed as percentages of pre-drug values. * indicates significant intragroup differences in fR between a-CSF condition and bumetanide application. # indicates significant intergroup differences in fR between PH and CONT or P0-1 and P3-4. *p < 0.05, ^**^p < 0.01, ^#^*p* < 0.05,^##^*p* < 0.01. C4 electrical activity of the 4th cervical ventral nerve root; IC4: integrated activity of the C4 ventral nerve root.

In the CONT group, 10 μM bumetanide–aCSF perfusion decreased fR during the last 5 min of exposure in MS preparations (*p* = 0.020; *n* = 12; Figures 1A,B) and from the sixth minute in PMS preparations (*F*_4,32_ = 7.176, *p* = 0.0003; *n* = 9; Figures 1C,D). In contrast, in the PH group, fR was not influenced by blockade of NKCC1 cotransporter in MS (*n* = 9, *p* = 0.61; Figures 1A,B) and PMS (*n* = 8, *p* = 0.12; Figures 1C,D) preparations. Although intragroup effects were different, normalised fR (in % of pre-bumetanide values) was similar in CONT and PH in both MS and PMS preparations (Figures 1E,F).

#### P3-4

In the CONT group, fR was not modified by blockade of NKCC1 cotransporter regardless of the preparation: MS (*p* = 0.21; *n* = 8; Figures 1A,B) and PMS (*p* = 0.053; *n* = 9; Figures 1C,D). In the PH group, exposure to bumetanide–aCSF did not change fR in the MS preparations (*p* = 0.46; *n* = 8; Figures 1A,B), but decreased fR in the PMS preparations (*F*_4,28_ = 3.068, *p* = 0.032; *n* = 8; Figures 1C,D) from the sixth minute of bumetanide–aCSF exposure. Normalised fR (in % of pre-bumetanide values) appeared to be lower in PH group than in CONT group in P3-4 PMS preparations (*p* = 0.012), but similar between CONT and PH in P3–4 MS preparations (Figures 1E,F).

#### P0-1 vs. P3-4

In the CONT group, the effect of age was present in PMS but not in MS preparations, fR was lower in P3-4 pups PMS preparations during the last 5 min under aCSF-bumetanide (*p* = 0.004; Figure 1F), than in P0-1 preparations. In PH pups, normalised fR was similar in both ages regardless of the preparation (Figure 1F).

### Abnormalities in Fictive Breathing Modulation by KCC2 Induced by Prenatal Hypoxia

#### P0-1

**FIGURE 2:**
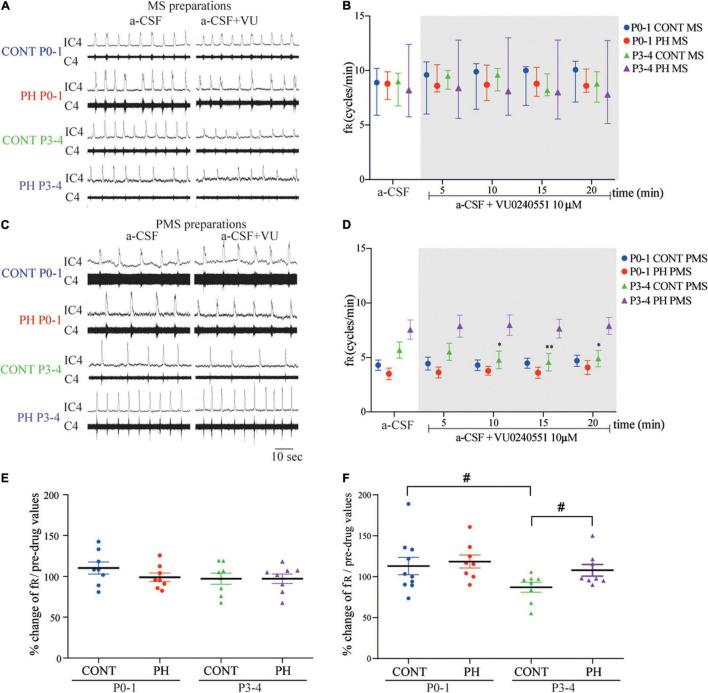
Impaired breathing related to KCC2 blockade was suppressed in prenatal hypoxic pup preparations. **(A,C)** Representative recordings of medulla **(A)** and ponto-medullary **(C)** preparations before (a-CSF) and during (a-CSF+VU) VU0240551 application, in controls (CONT) and prenatal hypoxic (PH) pups, at P0-1 and P3-4. **(B,D)** Frequency of fictive breathing before (a-csf) and every 5 min during (a-CSF + VU0240551 10 μM) VU0240551 application, in CONT and PH pups at P0-1 and P3-4. The fR is expressed as median [Q1; Q3] in medulla (MS) and as mean ± SEM in ponto-medullary- (PMS) preparations. **(E,F)** Scatter plots with surimposed mean ± SEM, illustrating changes in fR during the last 5 min of VU0240551 exposure, expressed as percentages of pre-drug values, in medulla **(E)** and ponto-medullary **(F)** preparations. *Indicates significant intragroup differences in fR between a-CSF condition and VU0240551 application. # Indicates significant intergroup differences in fR between PH and CONT or P0-1 and P3-4. *p < 0.05, ^**^p < 0.01, ^#^*p* < 0.05. C4 electrical activity of the fourth cervical ventral nerve root; IC4: integrated activity of the C4 ventral nerve root.

In the CONT group, 10 μM VU0240551 application had no influence on fR in MS (*p* = 0.71; *n* = 8; Figures 2A,B) and PMS preparations (*p* = 0.62; *n* = 10; Figures 2C,D). Same results were found in PH group, in which fR was unaffected in MS (*p* = 0.29; *n* = 8) and PMS preparations (*p* = 0.44; *n* = 8). There was therefore no difference in normalised fR (in % of pre-VU0240551 values) between MS and PMS in PH and CONT preparations during aCSF–VU0240551 exposure (Figures 2E,F).

#### P3-4

In the CONT group, 10 μM VU0240551 application had no influence on MS preparations (*p* = 0.83; *n* = 8; Figures 2A,B), but fR decreased from the sixth minute of KCC2 blockade in PMS preparations (*F*_4,28_ = 4.482, *p* = 0.0063; *n* = 7; Figures 2C,D). In contrast, in PH group, under VU0240551 exposure, fR was not modified in neither MS (*n* = 8, *p* = 0.46) nor PMS (*p* = 0.58; *n* = 8) preparations. Normalised fR (in % of pre-VU024055 values) was therefore lower in CONT than in PH (*p* = 0.043) in PMS preparations (Figure 2F).

#### P0-1 vs. P3-4

In MS preparations from both groups of animals, fR was similar with age during VU0240551 exposure, whereas in PMS preparations, a significant decrease in f_*R*_ occurred at P3-4 compared to P0-1 in CONT pups (*p* = 0.035; Figures 2E,F). This age change in KCC2 blockade was not observed in PH pups.

### Membrane Expression of Cl^–^ Cotransporters

**FIGURE 3:**
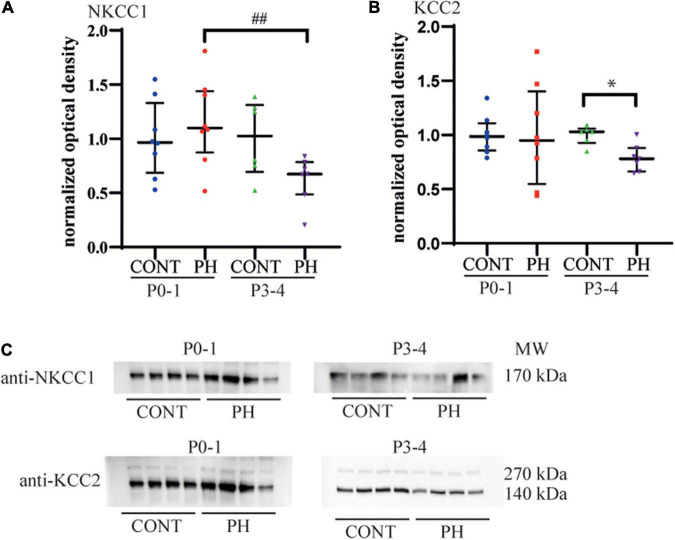
KCC2 expression decreased in the pons of prenatal hypoxic pups at P3-4, compared to age matched controls. **(A)** Expression of NKCC1 and **(B)** KCC2 in the pons at P0-1 and P3-4 in CONT and PH groups, expressed as normalised optical density. **(C)** Images showing Western blots of NKCC1 and KCC2 at P0-1 and P3-4 in CONT and PH pups. In PH pups, NKCC1 expression was lower at P3-4 than at P0-1. At P3-4, KCC2 expression was lower in PH than in CONT group. Scatter plots with surimposed median [Q1; Q3]. *p < 0.05 indicates significant differences between CONT and PH groups and ^##^ *p* < 0.01 indicate significant effect of age.

Since prenatal hypoxia disturbed both NKCC1 and KCC2 regulation of fictive breathing dependently from the pons, membrane expression of Cl^–^ cotransporters was analysed at this level. At P0-1, NKCC1 and KCC2 membrane expressions were similar in both groups, CONT and PH (*p* = 0.48 and *p* = 0.7, respectively; Figures 3A–C). By P3-4, although NKCC1 expression tended to be lower in PH pups than in CONT, there was no significant difference (*p* = 0.07; Figures 3A,C). At P3-4, KCC2 membrane expression was significantly decreased in the PH group in comparison with CONT pups at P3-4 (*p* = 0.019; Figures 3B,C). In PH pups, a significantly higher NKCC1 membrane expression was presented in P0-1 group compared to P3-4 group (*p* = 0.006; Figures 3A,C), and this effect of age was absent in CONT. The ratio NKCC1/KCC2 was similar in PH and CONT at both ages.

### Prenatal Hypoxia Increased Brain-Derived Neurotrophic Factor Levels in Brainstem

**FIGURE 4:**
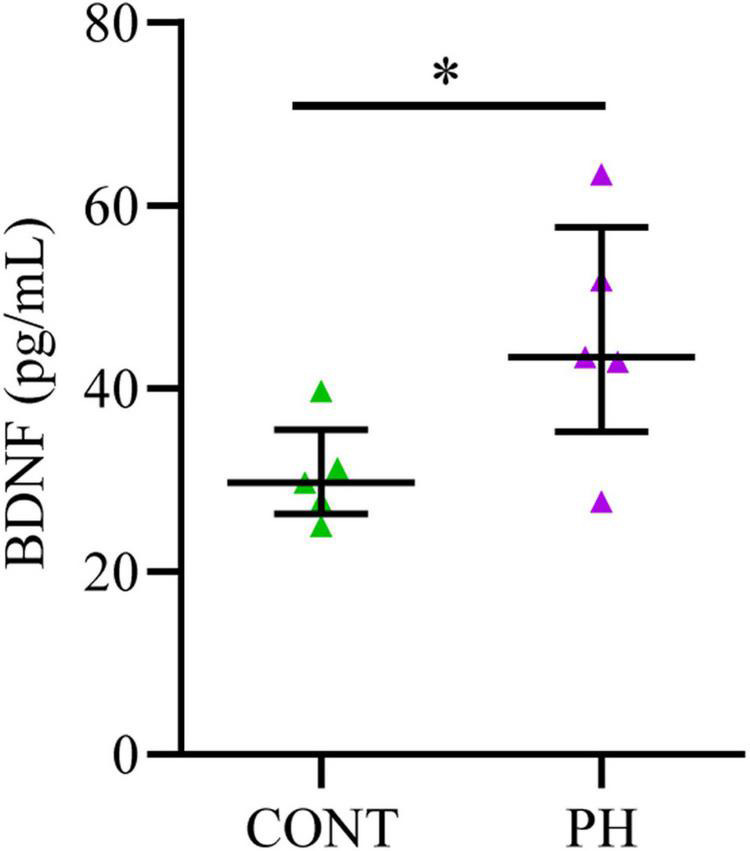
BDNF concentration increased in the pons of prenatal hypoxic pups, compared to control pups. Concentrations of BDNF evaluated by ELISA in the pons of P3-4 control and hypoxic pups expressed in pg/mL after tissue processing. Values are shown as median [Q1; Q3]. *p < 0.05 indicates significant differences between CONT and PH groups.

Since prenatal hypoxia induced a decrease in the membrane expression Cl^–^ cotransporters in the pons at P3-4, we quantified BDNF, a contributor of their regulation, in this region at that age. We noted a significant increase in BDNF content (*U = 3.5*; *p* = 0.031; Figure 4) in PH pups in comparison with CONT pups.

### Prenatal Hypoxia Increased the Number of Calpain-Positif Neurons in Brainstem Respiratory Structures

**FIGURE 5:**
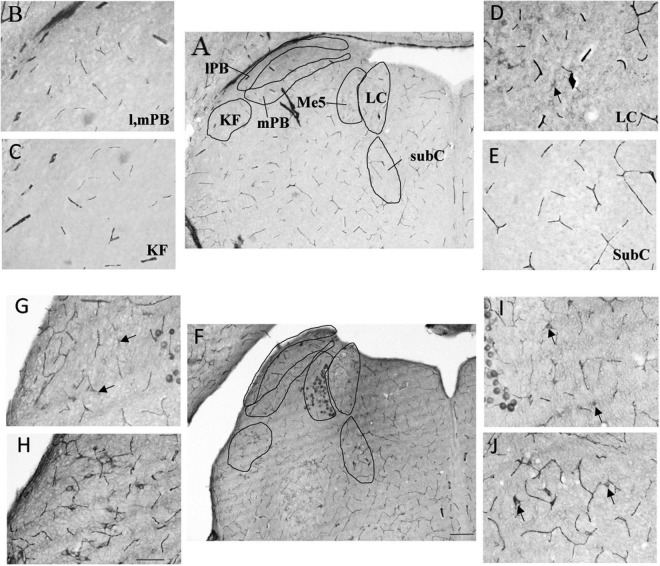
Expression of calpain was stronger in the pons of prenatal hypoxic pups than in controls. Photomicrography of labelling of calpain in breathing-related nuclei in the pons of PH **(A–E)** and CONT **(F–J)** pups. **(B–E)** Higher magnification of A (CONT). **(G–J)** Higher magnification of F (PH). lPB, lateral parabrachial nucleus; mPB, median parabrachial nucleus; KF, kolliker fuse nucleus; LC, locus coeruleus; subC, subcoeruleus. **(A,F)** Scale bar = 200 μm; **(B–E,G–J)** Scale bar = 100 μm. Black arrows point at calpain-expressing cells.

As prenatal hypoxia both supressed the decrease in fR induced by VU024055 and decreased the membrane expression of KCC2, we analysed distribution of calpain-positive neurons in pons aeras related to breathing. Locus Coeruleus and subcoeruleus nucleus had significantly more calpain-positive cells in PH pups (*p* = 0.0079 for each nucleus). The increase in calpain-positive cells was slight but significant in the Kolliker fuse (*p* = 0.031). We did not observe any difference in calpain positive cells in lateral and medial parabrachial nuclei (*p* = 0.31; Table 1 and Figure 5).

**TABLE 1:** Calpain expression in respiratory related areas of the pons in CONT an PH pups.

	CONT	PH
LC	0.3 [0.15,0.7]	4.6 [2.45,5.45] **
SubC	1.6 [0.5,2.95]	16.7 [10.8,21.35] **
lPB mPB	6.8 [1.35,10.3]	7.7 [4.85,18.95]
KF	1.7 [0.2,2.1]	4.4 [2.55,13.1] *

Values represent the number of calpain positive cells per section in PH and CONT pups at P4 (median with interquartile). LC, locus coeruleus; SubC, sub coeruleus; lPB, lateral parabrachial; mPB, median parabrachial; KF, Kölliker-fuse nucleus. Asterisks indicate significant differences between PH and CONT group, *p < 0.05 and **p < 0.01.

### Prenatal Hypoxia Disturbed the Modulation of Fictive Breathing Modulation by GABA_A_ or/and Glycine Receptors Throughout Differential Effects on Medulla Oblongata and Pons

Picrotoxin application on MS or PMS preparations induced tonic discharges, regardless of age and the presence of the pons, as previously shown (Shao and Feldman, 1997; Iizuka, 2003).

#### P0-1

**FIGURE 6:**
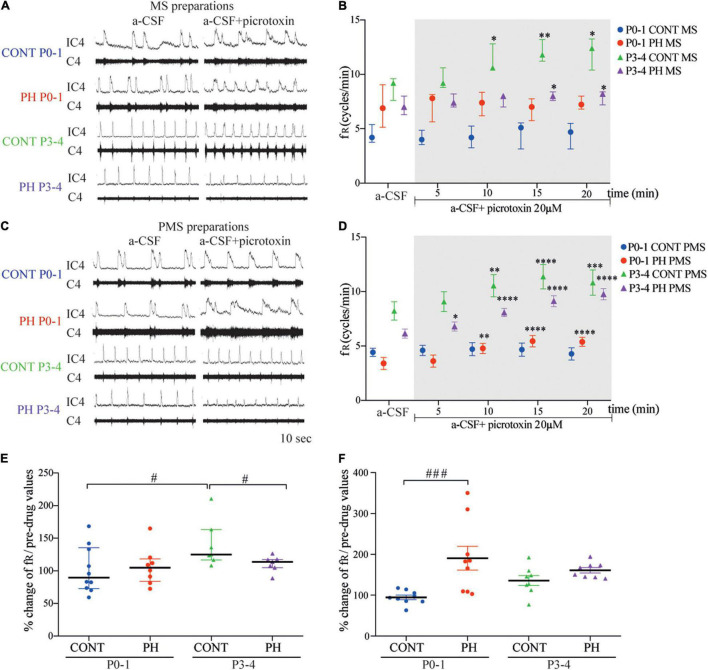
GABA_A_ receptor blockade increased fictive breathing frequency at P3-4 in all conditions. **(A,C)** Representative recordings of medulla **(A)** and ponto-medullary **(C)** preparations before (a-CSF) and during (a-CSF + picrotoxin) picrotoxin application, in control (CONT) and prenatal hypoxic (PH) pups, at P0-1 and P3-4. **(B,D)** Frequency of fictive breathing before (a-CSF) and every 5 min during (a-CSF + picrotoxin 20 μM) picrotoxin application, in CONT and PH pups, at P0-1 and P3-4. The fR is expressed as median [Q1; Q3] in medulla (MS) and as mean ± SEM in ponto-medullary (PMS) preparations. **(E,F)** Scatter plots with surimposed median [Q1; Q3] in medulla **(E)** and mean ± SEM in ponto-medullary **(F)** preparations, illustrating changes in fR during the last 5 min of picrotoxin exposure, expressed as percentages of pre-drug values. * indicates significant intragroup differences in fR between a-CSF condition and picrotoxin application. ^#^ indicates significant intergroup differences in fR between PH and CONT or P0-1 and P3-4. *p < 0.05, ^**^p < 0.01, ^***^p < 0.001, ^****^p < 0.0001, ^#^*p* < 0.05, ^#^*p* < 0.05, ^###^*p* < 0.001. C4 electrical activity of the 4th cervical ventral nerve root; IC4: integrated activity of the C4 ventral nerve root.

In CONT group, fR was not modified by picrotoxin exposure in neither MS (*p* = 0.58; *n* = 10; Figures 6A,B) nor PMS (*p* = 0.54; *n* = 9; Figures 6C,D) preparations, compared to that of the basal condition. In PH, fR of MS preparations also remained unchanged (*p* = 0.91; *n* = 8; Figures 6A,B) during exposure to picrotoxin. By contrast, fR was increased in PMS preparations of the PH group (*F*_4,32_ = 12.93, *p* < 0.0001; *n* = 8; Figures 6C,D) from 5 min during exposure to picrotoxin, compared to the basal condition. As a consequence, normalised fR (in % of pre-picrotoxin values) was higher in PMS preparations of PH pups than in CONT pups during the last 5 min of a-CSF–picrotoxin exposure (*p* = 0.0003; Figure 6F).

#### P3-4

In CONT group, picrotoxin–a-CSF perfusion enhanced the fR from the fifth minute of exposure in MS (*p* = 0.0043; *n* = 7; Figures 6A,B) and in PMS (*F*_4,28_ = 9.845, *p* < 0.0001; *n* = 8; Figures 6C,D) preparations, compared to basal conditions. In the PH group, blockade of GABA_A_ also induced an increase in fR in MS (*p* = 0.016; *n* = 7; Figures 6A,B) and PMS (*F*_4,28_ = 65.84, *p* < 0.0001; *n* = 8; Figures 6C,D) preparations. In MS preparations from PH pups, normalised fR (in % of pre-picrotoxin values) was lower than fR in CONT pups during the last 5 min of a-CSF–picrotoxin exposure (*p* = 0.041; Figure 6E).

In CONT pups, normalised fR under picrotoxin was higher at P3-4 than P0-1 in MS (*p* = 0.0107; Figure 6E) preparations. In PH pups, normalised fR (in % of pre-picrotoxin values) was similar at P0-1 and P3-4 group in MS (*p* = 0.92; Figure 6E) and in PMS (*p* = 0.23; Figure 6F) preparations during a-CSF-picrotoxin exposure.

### Prenatal Hypoxia and Glycine Receptors Blockade

#### P0-1

**FIGURE 7:**
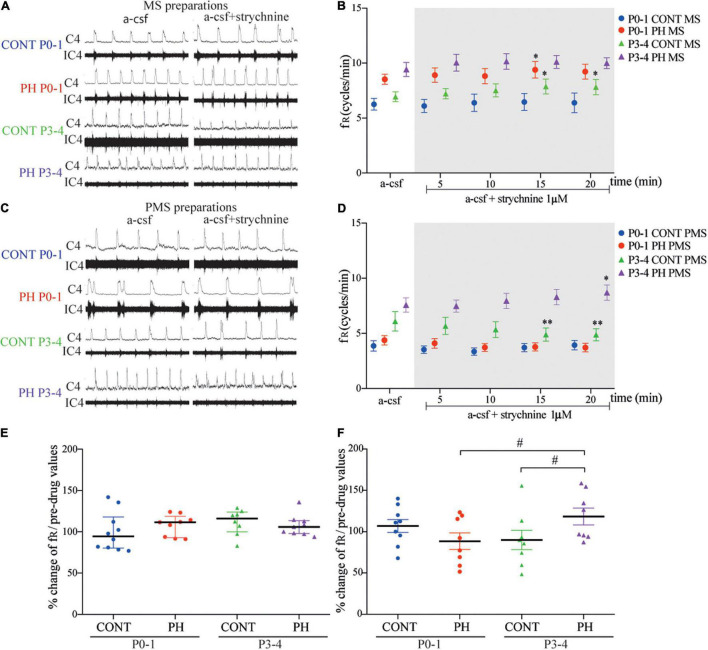
Glycine receptor channels blockade in prenatal hypoxic pup medullo-spinal preparation increased fictive breathing frequency but decreased in pontine-medullo-spinal preparation. **(A,C)** Representative recordings of medulla **(A)** and ponto-medullary **(C)** preparations before (a-CSF) and during (a-CSF +strychnine) strychnine application, in controls (CONT) and hypoxic (PH) pups, at P0-1 and P3-4. **(B,D)** Mean ± SEM of fictive breathing frequency before (a-CSF) and every 5 min during (a-CSF + strychnine 1 μM) strychnine application, in CONT and PH pups, at P0-1 and P3-4, in medulla (MS) and ponto-medullary (PMS) preparations. **(E,F)** Scatter plots with surimposed median [Q1; Q3] in medulla **(E)** and mean ± SEM in ponto-medullary **(F)** preparations illustrating change of fR during the last 5 min of strychnine exposure, expressed as percentage of pre-drug values. * indicates significant intragroup differences in fR between a-CSF condition and strychnine application. ^#^ indicates significant intergroup differences in fR between PH and CONT or P0-1 and P3-4. *p < 0.05, ^**^p < 0.01, ^#^*p* < 0.05. C4 electrical activity of the fourth cervical ventral nerve root; IC4: integrated activity of the C4 ventral nerve root.

In CONT group, strychnine had no effect on fR regardless of the preparations MS (*p* = 0.84, *n* = 10; Figures 7A,B) or PMS (*p* = 0.16, *n* = 9; Figures 7C,D). In the PH group, strychnine exposure increased fR slightly and temporarily during strychnine exposure between 11 and 15 min of the test in MS preparations (*F*_4,32_ = 2.943, *n* = 9, *p* = 0.035; Figures 7A,B), whereas no change was observed in the PMS preparations (*p* = 0.19; *n* = 8; Figures 7C,D). Normalised fR (in % of pre-strychnine values) was not different between the PH and CONT pups at this age.

#### P3-4

In the CONT group, glycine receptors blockade had an opposite effect on MS and PMS preparations. In MS preparations, fR was enhanced from the tenth min of exposure until the end of the test (*F*_4,28_ = 3.447, *p* = 0.02; *n* = 8; Figures 7A,B). In contrast, in PMS preparation, fR was decreased from the tenth minute until the end of strychnine–aCSF exposure (*F*_4,28_ = 4.598, *p* = 0.005; *n* = 8; Figures 7C,D). In the PH group, strychnine–aCSF application had no impact on fR in MS preparations (*p* = 0.11; *n* = 9; Figures 7A,B). In PMS preparations, the ANOVA repeated measure test followed with a *post hoc* test indicated a significant increase in fR in the last 5 min of the strychnine exposure (*F*_4,28_ = 2.887, *p* = 0.040; Figures 7C,D). Normalised fR (in % of prestrychnine values) was significantly higher in the PH group than in the CONT group during the last 5 min of strychnine exposure in PMS preparations (*p* = 0.011; Figure 7F).

During blockade of glycine receptors, normalised fR (in % of pre-strychnine values) remains similar in MS preparations in P0-1 and P3-4 pups in CONT (*p* = 0.27) and in PH pups (*p* = 0.76; Figure 7E). Unlike in PH group, a significant increase in normalised fR (in % of pre-strychnine values) was observed in PMS preparations in P3-4 pups in comparison with P0-1 pups (*p* = 0.026; Figure 7F), whereas age had no effect in CONT pups in PMS preparations.

## Discussion

Our study focused on the impact of prenatal hypoxia on Cl^–^-dependent regulation of central respiratory drive. Interests surrounding this question arose from the following observations: (1) alterations of chloride homeostasis related to a change in membrane expression in NKCC1 and KCC2, which can result from environmental insults, including prenatal stress, (2) prenatal hypoxia is known to induce neurodevelopmental abnormalities, and (3) central respiratory drive, one of the earliest functions to develop in foetus, which is sensitive to prenatal hypoxic stress. Through analysing fictive breathing on *ex vivo en bloc* preparations under pharmacological applications, we showed that prenatal hypoxia disrupts NKCC1 and KCC2 Cl^–^-dependent regulation of central respiratory drive and influences GABA/GABA_A_ and glycine systems whose cell effects depend on intracellular Cl^–^ concentration. Using western blot, ELISA test, and immunohistochemistry, we demonstrated that these functional data were closely linked to a pontin decrease in membrane expression of Cl^–^ cotransporters, an increase in BDNF in the pons, and an enhanced number of calpain-positive neurons in nuclei related to breathing.

### Age-Dependant Effect of Central Respiratory Drive Modulation by NKCC1/KCC2 in the First Days of Postnatal Life

Our pharmacological results revealed changes in chloride homeostasis in the first few days after birth which led to changes in CRD modulation. For instance, blockade of NKCC1 decreased fR at P0-1 but produced no effect at P3-4. By contrast, KCC2 did not seem to modulate CRD at birth, but its blockade at P3-4 decreased fR when the pons was included in the preparation. Thus, chloride homeostasis strongly depends on NKCC1, but not on KCC2, in the respiratory network cells at P0-1, whereas opposite effects were found at P3-4. Such observations are consistent with data from the literature showing an age-dependent change in the effects of chloride-mediated conductances on fR. In rat brainstem–spinal cord preparations taken between E17 embryonic day and P2, a pretreatment with bumetanide was made in which the NKCC1 inhibitor, used in the present study, abolished the chloride-dependant GABA_A_ respiratory effect, whereas the inhibition of KCC2 by furosemide had no significant effect before E18 but decreased fR in neonatal preparations (Ren and Greer, 2006). Thus, in addition to the impact on CRD of chloride cotransporters in the transition from foetus to newborn, our data showed that the CRD was strongly influenced by the maturation of chloride homeostasis during the first few days of postnatal life. Concerning the disappearance of respiratory modulation by NKCC1, membrane expression of NKCC1 in the medullary respiratory neurons did not vary within the first week of postnatal life (Liu and Wong-Riley, 2012; Wong-Riley et al., 2019). Maturation of its respiratory effect must, therefore, not depend on a generalised decrease in expression in respiratory cells but rather on its functionality. In such a context, we did not observe any significant decrease in NKCC1 membrane expression between P0-1 and P3-4. It is likely that maturation of respiratory modulation by NKCC1 within the first few days of life depends on the regulation of its phosphorylated form as already shown (Watanabe and Fukuda, 2015). On the implementation of CRD modulation by KCC2, our data suggest that maturation of KCC2 takes place heterogeneously within the respiratory network since we observed the appearance of a modulation of CRD at P3-4 only in presence of the pons. To our knowledge, our study is the first to discriminate medulla oblongata- or pons-dependent influences on the respiratory modulation by KCC2 in the first few days of life. While histological data have shown a gradual increase in KCC2 expression in cells of different medullary respiratory neuronal groups continuous from P0 to P10 (Wong-Riley et al., 2019), there are no data at the pontine level. In the medulla oblongata, overall KCC2 expression in respiratory nuclei reached maximum at P10 with, for example, an increase in preBötzinger complex and parafacial respiratory group by around 50% of P0 expression at P4 and 100% at P10 (Wong-Riley et al., 2019). Since we observed no effect of KCC2 blockade on CRD at P3-4 in medullary-spinal cord preparations, it is likely that KCC2 were either not addressed to the membrane or not functional. As we observed a respiratory impact in presence of the pons, we hypothesised that KCC2 maturation is more advanced in the pons at P3-4 than in the medulla oblongata in regard to respiratory regulation. Since our data suggest that there is no difference in the membrane addressing of KCC2 between P0-1 and P3-4, we hypothesised that the CRD modulation by KCC2 involving pons is linked to protein regulation, as we suggested for NKCC1 (Kelsch et al., 2001; Watanabe and Fukuda, 2015).

### Prenatal Hypoxia Led to a Delayed Pons-Dependent Maturation of NKCC1/KCC2 Cl^–^ Cotransporters in the Modulation of Central Respiratory Drive

Pharmacological blockade of Cl^–^ cotransporters in *ex vivo* preparations of PH newborn rats suggests that prenatal hypoxia disturbs the pons-dependent maturation of chloride CRD modulation which takes place within the first days of postnatal life. First of all, at P0-1, NKCC1 did not regulate fR in PH preparations in contrast to the regulation observed in CONT pups and at P3-4. NKCC1, despite a drop in his expression with age, still regulated the CRD in PH preparations with the pons, which was not the case at this stage in CONT. Second of all, at P3-4, although normal maturation led to a CRD regulation by KCC2 that involves pontine areas, this regulation was not observed in PH preparations. All together, these observations suggest a pivotal role of the pons in the effect of prenatal hypoxia on chloride CRD modulation within the first few days of life. Our data regarding the membrane expression of Cl^–^ cotransporters suggest a different effect of prenatal hypoxia on the two types of chloride transporters; although the pontine NKCC1 membrane expression was similar in CONT and PH, the membrane expression of KCC2 decreased in the pons of PH pups at P3-4. Unlike what was discussed concerning normal development, regulation of membrane expression of NKCC1 might be responsible for the effect of prenatal hypoxia on CRD regulation as well as regulations of the functional state of NKCC1 through dynamic regulation, such as phosphorylation variations (Watanabe and Fukuda, 2015). Concerning KCC2, membrane expression regulation seems to be involved. In fact, prenatal hypoxia ischemia induced a decrease in the expression of KCC2 related to an excess of calpain activation. This protein mediated the cleavage of KCC2 (Jantzie et al., 2016; Plantier et al., 2019). Higher levels of BDNF and an increase in calpain content in pontin respiratory nuclei observed in PH pups suggest the involvement of these underlying contributors. BDNF *via* activation of its high affinity receptor, TrkB, has indeed decreased KCC2 transcript and protein expression as shown in other physiological contexts (Rivera et al., 2002). Calpains, cystein intracellular proteases activated by intracellular Ca^2+^ or by BDNF and its receptor TrkB, lead to cleavage of KCC2 (Zadran et al., 2010; Puskarjov et al., 2012; Plantier et al., 2019). Among the pontin respiratory structures impacted by delayed maturation of KCC2, we hypothesised that Kolliker–Fuse nucleus that displayed an increase in calpain-positive neurons played a pivotal role in the alterations of CRD Cl^–^ regulation because of their recognised role in the CRD as the “pontin respiratory group” (Ikeda et al., 2017). It should also be noted that our histological data suggest an impact of prenatal hypoxia on two pontine structures that participate in the adaptation of CRD during hypoxic or hypercapnic challenges: the locus coeruleus and the subcoeruleus nucleus (Coates et al., 1985; Breen et al., 1997; Oyamada et al., 1998; Nitsos and Walker, 1999; Walker et al., 2000; Joubert et al., 2016). Such an observation may suggest that the disregulation of KCC2 induced by prenatal hypoxic in locus coeruleus and subcoeruleus could alter the ability to regulate CRD in a context of gas challenges.

### Prenatal Hypoxia Altered the Regulation of Central Respiratory Drive by GABA/GABA_A_ and Glycine

#### Developmental Aspect of the GABA/GABA_A_ and Glycine Impact on Central Respiratory Drive

Based on our results, GABA and glycine do not seem essential to newborn rhythmogenesis, but seem crucial for the shaping and modulating of the overall respiratory network output. In P0-1 group of normoxic pups there was no change in fictive breathing frequency in medulla or ponto-medullary preparations during GABA_A_ or glycine receptors blockade. Nevertheless, in P3-P4 group, medulla or ponto-medullary preparations exhibited an increase in fictive breathing when picrotoxin was applied, compared to the baseline condition. This observation supports the hypothesis that GABA became “inhibitory” between P0-1 and P3-4. It has been shown that bicuculline application, a GABA_A_ antagonist, increased C4 burst frequency in medulla preparation from P0-4 rat pups (Kato et al., 2000), but no exact detail was given regarding a possible variation between ages. Moreover, in a study using mouse medulla slice, blockade of GABA_A_ receptors did not alter rhythmic bursts in the XIIn between P0-P2, but increased this activity in slices from P4-P6 pups (Ritter and Zhang, 2000), thus confirming the postnatal set-up of the GABA inhibitory role. We found similar results when glycinergic receptors were blocked, strychnine had no effect at P0-P1 but rather at P3-P4. One could enhance or depress fictive breathing differentially according to the absence or the presence of the pons in preparations which supports the idea that glycinergic systems, such as the GABAergic system, undergo drastic changes after birth. It also highlights the role of the pons in the central respiratory command, and how it changes after birth. We then focused on the comparison between preparations with and without the pons in order to better understand its function and how GABA and glycine are involved.

#### Abnormal Development of GABA/GABA_A_ and Glycine Inhibition of Central Respiratory Drive After Prenatal Hypoxia

In P0-1 group of normoxic pups, we did not observe any difference in fictive breathing frequency in medulla or ponto-medullary preparations during GABA_A_ or glycine receptors blockade. However, P0-1 preparations with or without pons coming from PH pups exhibited significant differences. The fictive breathing frequency was increased during GABA_A_ blockade in ponto-medullary preparations and during glycine receptors blockade in medullary preparations. Since the fR in the PMS preparation was lower in PH pups than the fR in CONT groups, and since GABA_A_ receptor blockade increased the fR in PH but not in CONT, it is possible that prenatal hypoxia leads to increased efficiency of the GABAergic system on CRD.

The stimulating effects of glycine have already been described in studies where prenatal medulla preparations were exposed to nicotine. Direct glycine exposure was made and it showed fictive breathing depression in rat medulla preparations, compared to control preparations. Depression was accentuated by exposure to glycine baths and was abolished using strychnine (Luo et al., 2004, 2007). Interestingly, from P0-1 we obtained an effect of strychnine in PH preparations which was not detectable in CONT pup preparations, which suggests that a glycinergic modulation of the respiratory network in the medulla was altered by the prenatal hypoxia. In fact, strychnine exposure can lead to a difference in loss between PH and CONT preparations. Concerning the effect of GABA_A_ blockade, picrotoxin induced an increase in fictive breathing frequency in ponto-medullary PH pup preparations, but not in the CONT group. This result suggests a change in the GABAergic pontine influence of medullary respiratory network after prenatal hypoxia. Muscimol, an agonist of GABA_A_ receptor, has been shown to enhance the depression of fictive breathing frequency or apnea duration in medulla-spinal cord preparations (Luo et al., 2004, 2007). Our results indicate that prenatal hypoxia had no impact on GABAergic modulation of breathing at the medulla level, but modified GABAergic pontine modulation of medullary respiratory network.

The influences of activation by GABA_A_ or glycine receptors on breathing network change with maturation (Paton and Richter, 1995; Ritter and Zhang, 2000; Zhang et al., 2002; Ren and Greer, 2006; Okabe et al., 2015). In P3-4 aged pups, the GABA_A_ antagonist increased fictive breathing frequency in the two groups regardless of the preparations. While glycine receptor blockade increased fR in medulla preparation and decreased it in ponto-medullary preparation of CONT pups, prenatal hypoxia led to different results as follows: in medulla preparations, strychnine had no influence but it slightly enhanced fictive breathing frequency in the ponto-medullary preparations at the end of the test. The changes in the influence of the blockade of GABA_A_ or glycine between normoxic and PH pups on fictive breathing frequency might be related to the differential maturation of NKCC1 and KCC2 cotransporters in the pons or medulla. In normoxic pups, bumetanide had no effect on both preparations; whereas it decreased fR in ponto-medullary preparations of the PH pups, and had no influence on the medullary preparations. The KCC2 cotransporters inhibition with VU0240551 had no effect in PH pups in the two preparations, but it decreased fictive breathing frequency in ponto-medullary preparations with no effect on medullary preparations in CONT pups. Results in CONT pups are concordant with literature. Before P1, pretreatment with bumetanide abolished the influence of muscimol on breathing, while the blockade of KCC2 cotransporters was ineffective. However, it became effective after P1 and either attenuated or abolished the effect of muscimol (Ren and Greer, 2006) or decreased the XII burst frequency in mouse (Okabe et al., 2015). Furthermore, additional experiments could investigate whether prenatal hypoxia modulates the number of GABA and glycine receptors. If this were the case, such modulations could contribute to the alterations highlighted in this study.

## Conclusion

In conclusion, the present study highlights impact of Cl^–^ cotransporter disruption caused by prenatal hypoxia on the respiratory drive by pointing out the involvement of pontin neural groups and GABA/GABA_A_ and glycin neurotransmission systems. Our data also suggest that pharmacological paradigms aimed to reduce BDNF, or that calpain levels could represent promising strategies to normalise KCC2 levels in the context of prenatal hypoxia, or high risks for the emergence of neurodevelopmental disorders, a medical field where there are little pharmacological preventive approaches.

## Data Availability Statement

The raw data supporting the conclusions of this article will be made available by the authors, without undue reservation.

## Ethics Statement

The animal study was reviewed and approved by the INT Neurosciences Ethic Committee No. 71 for Animal Research.

## Author Contributions

CC contributed substantially to acquisition and analysis of electrophysiological and pharmacological data and manuscript preparation. AC contributed substantially to acquisition and analysis of immunohistochemical data, statistical analysis of electrophysiological data, and manuscript preparation. J-OC review of manuscript. SL and CB contributed substantially to acquisition of Western Blots. LB contributed substantially to data interpretation, literature search, discussion of results and implications, and writing of the manuscript. JP contributed substantially to study design and manuscript preparation. FC contributed substantially to study design, data interpretation, literature search, analysis of data, and writing of the manuscript. All authors are accountable for all the aspect of the work.

## Conflict of Interest

The authors declare that the research was conducted in the absence of any commercial or financial relationships that could be construed as a potential conflict of interest.

## Publisher’s Note

All claims expressed in this article are solely those of the authors and do not necessarily represent those of their affiliated organizations, or those of the publisher, the editors and the reviewers. Any product that may be evaluated in this article, or claim that may be made by its manufacturer, is not guaranteed or endorsed by the publisher.
